# Tetraspanin CD9 determines invasiveness and tumorigenicity of human breast cancer cells

**DOI:** 10.18632/oncotarget.3419

**Published:** 2015-02-11

**Authors:** Germana Rappa, Toni M. Green, Jana Karbanová, Denis Corbeil, Aurelio Lorico

**Affiliations:** ^1^ Cancer Research Center, Roseman University of Health Sciences with Roseman University College of Medicine, Las Vegas, NV, USA; ^2^ Tissue Engineering Laboratories (BIOTEC) and DFG Research Center and Cluster of Excellence for Regenerative Therapies Dresden, Technische Universität Dresden, Tatzberg, Dresden, Germany

**Keywords:** breast cancer, CD9, cell invasion, plasma membrane protrusions, tetraspanin

## Abstract

Interaction of breast cancer cells (BCCs) with stromal components is critical for tumor growth and metastasis. Here, we assessed the role of CD9 in adhesion, migration and invasiveness of BCCs. We used co-cultures of BCCs and bone marrow-derived multipotent mesenchymal stromal cells (MSCs), and analyzed their behavior and morphology by dynamic total internal reflection fluorescence, confocal and scanning electron microscopy. 83, 16 and 10% of contacts between MDA-MB-231 (MDA), MA-11 or MCF-7 cells and MSCs, respectively, resulted in MSC invasion. MDA cells developed long magnupodia, lamellipodia and dorsal microvilli, whereas long microvilli emerged from MA-11 cells. MCF-7 cells displayed large dorsal ruffles. CD9 knockdown and antibody blockage in MDA cells inhibited MSC invasion by 95 and 70%, respectively, suggesting that CD9 is required for this process. Remarkably, CD9-deficient MDA cells displayed significant alteration of their plasma membrane, harboring numerous peripheral and dorsal membrane ruffles instead of intact magnupodium/lamellipodium and microvillus, respectively. Such modification might explain the delayed adhesion, and hence MSC invasion. In agreement with this hypothesis, CD9-knockdown suppressed the metastatic capacity of MDA cells in mouse xenografts. Our data indicate that CD9 is implicated in BCC invasiveness and metastases by cellular mechanisms that involve specific CD9^+^ plasma membrane protrusions of BCCs.

## INTRODUCTION

The formation of breast cancer metastases requires an interaction in the tumor microenvironment between malignant epithelia and local stroma [1, 2]. In order to adhere to, communicate with, and transfer materials to/from stromal cells, breast cancer cells (BCCs) often employ different types of plasma membrane protrusions (PMPs), including filopodia and lamellipodia [3]. Some of these PMPs are also instrumental for BCCs to spread and reach distant sites, helping them to move between, and invade, stromal cells. In addition to passing through intercellular gap junctions, BCCs can use a transcellular route for intra/extra-vasation, essential steps of the metastatic process [4]. Cancer cell invasion is also at the basis of *entosis*, cell-in-cell structures observed in a variety of tumors [5], and of the formation of hybrid cancer cells by cell-cell fusion, which contributes to aneuploidy and aberrant gene expression patterns [6-11]. Tetraspanin-29 (CD9, motility-related protein-1) is involved in the metastatic process and depending on the context as a metastasis suppressor or promoter [12]. Physiologically, it is involved in cell fusion, adhesion, motility, proliferation, and signaling [13] and is required for egg-sperm fusion leading to fertilization [14, 15]. CD9 has also an important role in muscle cell fusion [16] and in canine distemper virus and HIV-1-induced cell-cell fusion [17, 18]. Based on its physiological importance and its putative involvement in cancer metastasis, we sought to investigate whether CD9 has a role in the cellular interaction between human BCCs and bone marrow-derived multipotent mesenchymal stromal cells (MSCs). We found that CD9 is expressed on diverse types of PMPs of BCCs, and is required for MSC invasion. CD9 disruption resulted in profound alterations of plasma membrane of BCCs *in vitro*, including the impaired formation of magnupodium, lamellipodium and microvillus. Upon transplantation of CD9-deficient cells in a murine model, an inhibition of tumor growth was observed after one week as well as a partial loss of metastatic capacity. These novel CD9 functions could be exploited to develop innovative breast cancer therapeutic strategies.

## RESULTS

### Differential expression of CD9 among BCC lines

**Figure 1:**
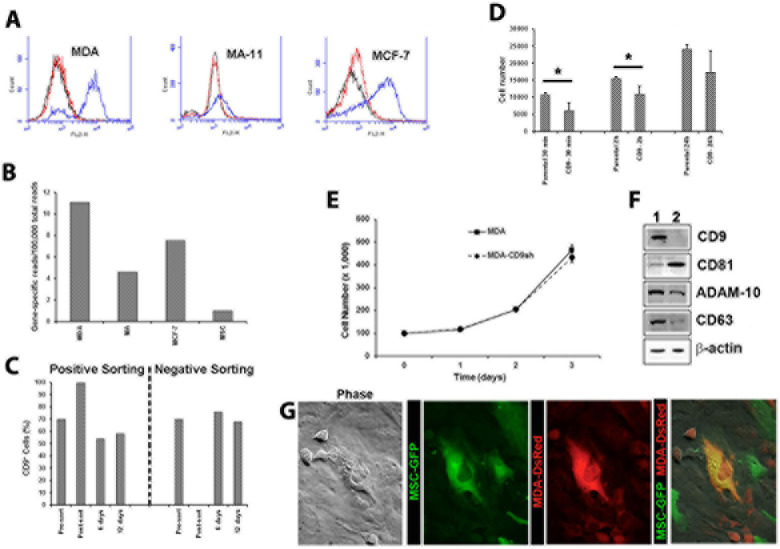
Expression and functions of CD9 in BCCs **A.** Flow cytometric analysis of MDA, MA-11, MCF-7 (blue) and CD9shRNA (red) cells labeled with PE-conjugated anti-CD9 mAb. Black, parental cells labeled with PE-conjugated isotype. **B**. RNA sequencing data for CD9 in MDA, MA-11 (MA), MCF-7 cells and MSCs. Data are expressed as gene-specific CD9 mRNA reads per 100,000 total reads. **C**. MDA cells were labeled with PE-conjugated anti-CD9 Ab and sorted by FACS. CD9-positive and negative cell fractions were cultured for 6 and 12 days, and the cell surface CD9 expression was re-assessed using another anti-CD9 Ab against a distinct CD9 epitope. **D**. The CD9 knockdown in MDA cells causes a delay in the adhesion to plastic substratum. Cells (2.5 × 10^4^) were plated in tissue culture-treated plastic dish and the number of adherent cells was evaluated after 30 min, 2 h and 24 h. *p<0.05. **E.** Growth curves of MDA and MDA CD9shRNA (MDA-CD9sh) cells for a period of three days. **F**. Parental (lane1) and CD9 shRNA (lane 2) MDA cells were probed by immunoblotting for different proteins as indicated. Actin was used as a loading control. **G**. DsRed-transduced MDA cells were co-cultured with GFP-transduced MSCs for 5 days and dual fluorescent cells were sorted. Occasional phenomena of entosis were observed 24 h after plating. Scale bars, 20 μm.

In this study, we used three distinct cell lines to investigate the implication of CD9 in BCC invasiveness and tumorigenicity. Its cell surface expression was monitored by flow cytometry. MDA-MB-231 and MCF-7 BCCs displayed a heterogeneous expression of CD9 which comprised 74, 19 and 59% of total cells, respectively (Fig. 1A, blue). The differential CD9 expression among cell lines is also observed at mRNA levels (Fig. 1B). Although the underlying cause of such heterogeneous expression is unknown, we observed conversion in culture of CD9^+^ into CD9^−^ cells and vice versa. Indeed, upon sorting of CD9^+^ and CD9^−^ MDA cell fraction (>99.8 % pure) from an exponential culture growth and their subsequent culture for 6 or 12 days, both cell fractions reformed mixed population with a ratio similar to parental MDA cells (Fig. 1C).

### CD9 is required for invasion of BCCs into MSCs

**Figure 2:**
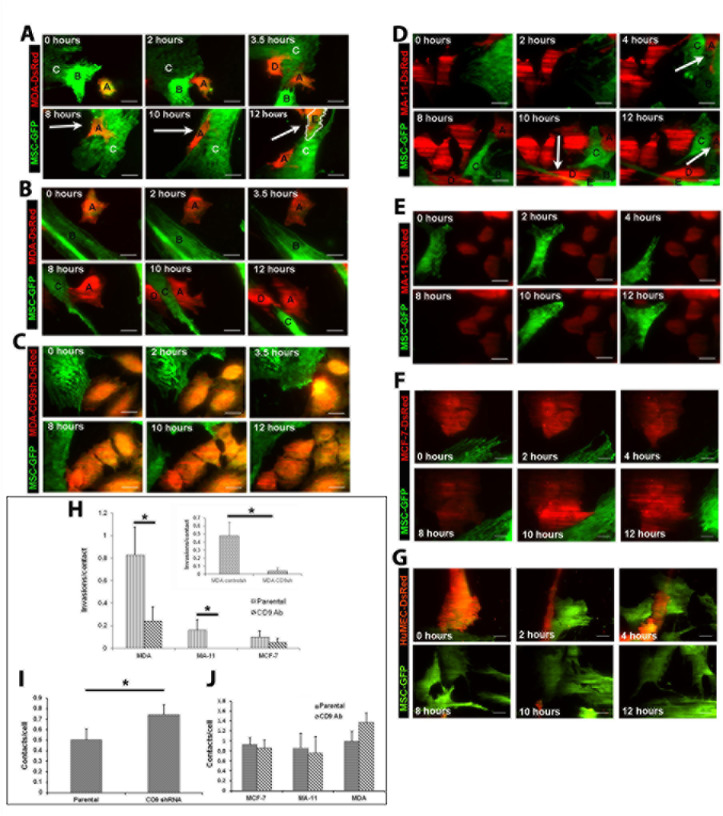
CD9-dependent invasion of MDA or MA-11 cells into MSCs **A.** Representative TIRF time-lapse images showing the partial/complete invasion, egress and trans-cell migration of MDA-DsRed cells into GFP-transduced MSCs over 12 h period. MDA-DsRed cell *A* fully invaded MSC *C* by 8 h (arrow), and remained within the next four hours; MDA-DsRed cell D transiently entered the observation field at 3.5 h; MDA-DsRed cell *E* (outlined in white) entered the observation field between 10 and 12 h, and was seen within the same MSC at 12 h. **B**. An anti-CD9 Ab was added to the MDA-DsRed/MSCs-GFP cells, which were recorded as described in panel A. Contacts and partial entry of MDA cells into MSCs were observed, but not invasion. MDA-DsRed cell *D* entered the observation field between 10 and 12 h. **C** MDA CD9 shRNA cells plated with MSCs-GFP. Contacts and partial entries of DsRed-labeled CD9-deficient MDA cells were observed, but not invasion. **D**. Representative TIRF time-lapse images showing the entry of MA-11-Ds-Red into MSCs-GFP. At 4 h, a MA-11 cell (*A*) invaded a MSC (*B*). Between 8 and 12 h, a second MA-11 cell (*D*) invaded and passed through MSC *E*. **E.** An anti-CD9 Ab was added to the MA-11-Ds-Red/MSCs-GFP cells. Only cell contacts were observed. Note that MSC-GFP cell observed at 4 hours is leaving transiently the field of observation at 8 hours. **F**. **G**. MCF-7-DsRed (F) or HuMEC-DsRed (G) cells were cultured with MSCs-GFP and TIRF images were recorded at indicated time points over 12 h period. No full cell invasions were observed. **H** Quantification of the invasion events of BCCs into MSCs per cell contact measured by TIRF in the absence (parental) or presence (CD9 Ab) of anti-CD9 mAb in the cultured medium. Inset, invasions per contact of MDA control shRNA (controlsh) and MDA CD9shRNA (CD9sh) cells. * p < 0.05. I Average number of contacts between parental or CD9-deficient MDA cells and MSCs. * p < 0.05. **J.** Addition of anti-CD9 Ab did not change the frequency of intercellular contacts between BCCs and MSCs. B-E, Red, DsRed; green, GFP. Scale bars, 25 μm.

To investigate the CD9 role in BCC invasiveness, we co-cultured BCC lines with MSCs and monitored their migratory behavior by time-lapse total internal reflection fluorescence (TIRF) and confocal imaging. MSCs were derived from pooled bone marrow samples of healthy adult donors, and cultured on poly-L-lysine as a substratum [19]. To visualize them, BCCs and MSCs were transduced with DsRed- and GFP-based retroviral vectors, respectively. In 1:1 ratio co-cultures of DsRed-expressing MDA, MA-11 or MCF-7 cells with MSCs-GFP, we observed that 83% of direct contacts between MDA-MSC resulted in MSC invasion, whereas only 16 and 10% of MA-11-MSC and MCF-7-MSC contacts lead to an invasion (Fig. 2A, D, F, H; Supplementary Video S1). Since the evanescent TIRF wave penetrates only 100-150 nm into the sample [20], overlapping fluorescence (green and red) signal did not derive from cell stacking, but from cell-cell invasion. Under same conditions, no invasion of human mammary epithelial cells (HuMECs) into MSCs was observed (Fig. 2G).

Next, we targeted the expression of CD9 by lentiviral-mediated short-hairpin RNA (shRNA) transduction using a pool of three distinct anti-CD9 shRNAs. Its expression was abrogated in MDA, MA-11 and MCF-7 cell lines as observed by flow cytometry (Fig. 1A). Upon CD9 knockdown, we found a 10-fold increase in the expression of CD81, a tetraspanin protein-interacting partner of CD9, in MDA CD9shRNA cells (Fig. 1F) [21, 22]. CD81 could potentially compensate for the lack of CD9 as reported in other cellular systems including knockout models, and consequently attempt to rescue the CD9 deficient phenotype [23, 24]. 5- and 3-fold decreases in the expression of tetraspanin CD63 and ADAM10, respectively, were observed (Fig. 1F). ADAM10 is a sheddase that cleaves Her2 (CD340), of which the latter oncogene plays a role in the development and progression of aggressive breast cancers [25, 26]. The abrogation of CD9 expression neither affects the growth rate (Fig. 1E) nor the clonogenic ability of MDA cells (clonogenicity of 41.5 +/− 1.3% and 42.1 +/− 2.2% for MDA and MDA CD9 shRNA cells, respectively). Remarkably, MSC invasion rates of CD9-deficient MDA cells were significantly inhibited (Fig. 2C; Supplementary Video S2). Such phenomenon was not a consequence of the reduction of BCC-MSC contacts, since CD9-deficient cells showed more contacts with MSCs than parental cells (Fig. 2I). Finally, the invasiveness of MDA or MA-11 cells was evaluated using anti-CD9 blocking antibody (Ab). Again, MDA and MA-11 invasiveness were significantly inhibited (Fig. 2B, E, H), although the interactions of BCCs and MSCs were unchanged (Fig. 2J).

### Implication of CD9 in BCC morphology and adhesion

**Figure 3:**
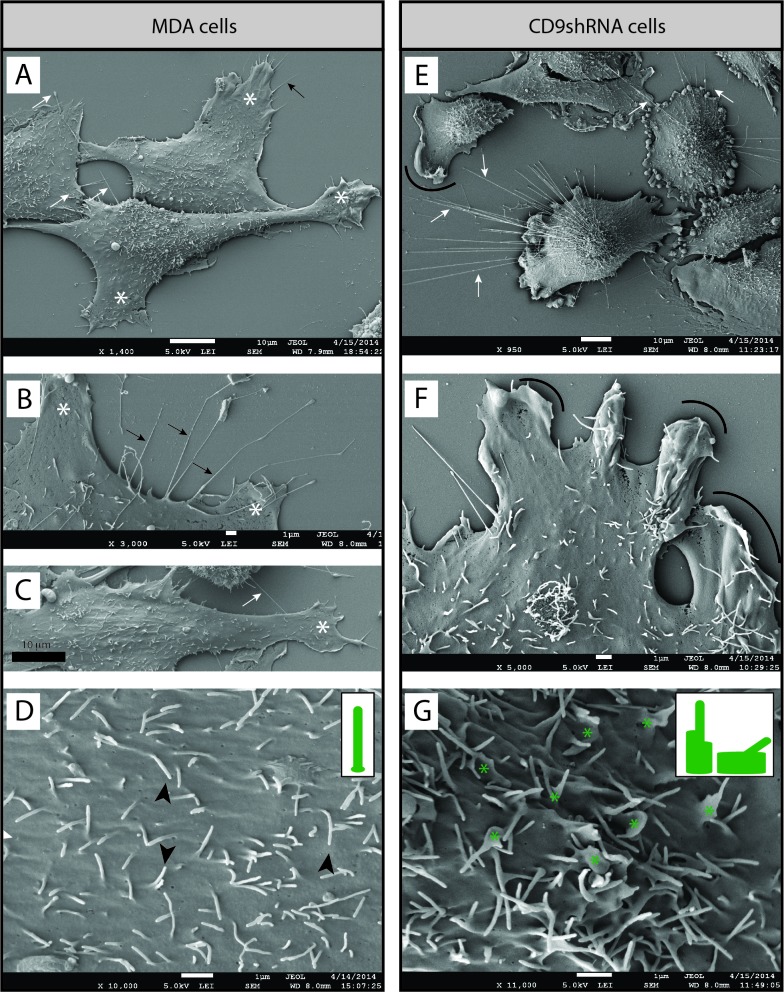
CD9 influences BCC morphology **A-G**. MDA cells (A-D) and their CD9-shRNA-transduced counterpart (E-G) grown on poly-L-lysine-coated coverslips were analyzed by SEM. Various types of PMPs such as lamellipodium (A-C, white asterisks), filopodium (A-B, black arrows), and dorsal microvillus-like structures (D, black arrowheads) were identified on MDA cells. Membrane ruffles were detected at the edge (E, F, arcs) or dorsal part (G, green asterisks) of CD9-deficient cells. Thin membrane processes emerged from the dorsal side of parental and CD9-deficient cells (A, C, E, F, white arrows). The distinct shapes of microvillus-like structures or membrane ruffles/altered microvillus-like structures observed at the apex of cells are depicted in the insets (D, G, respectively). Scale bars are indicated.

To gain more insight into the morphology of BCCs and the impact of CD9 on their structures, we analyzed them by scanning electron microscopy (SEM). The characteristics of different types of PMPs generated by MDA, MDA CD9 shRNA, MCF-7 and MA-11 cells are summarized in Table 1. while parental MDA cells were spread and created lamellipodia and filopodia close to the poly-L-lysine substratum, or interestingly, above the neighboring cells (Fig. 3A-C, white asterisks and black arrows, respectively; Supplementary Fig. S1), CD9-deficient cells displayed a certain impairment in adhesion, particularly at their cell border where numerous membrane ruffles were observed (Fig. 3E, F, arcs; Supplementary Fig. S1, arcs). As a consequence, the knockdown of CD9 resulted in delayed adhesion of MDA cells as well as MA-11 and MCF-7 to poly-L-lysine (not shown) or tissue culture-treated plastic substratum (Fig. 1D). In addition, the knockdown of CD9 caused severe modifications of the plasmalemma of MDA cells. While microvillus-like structures covered the apex of parental MDA cells (Fig. 3D, black arrowheads; Supplementary Fig. S1), they were rarely observed in an intact form in MDA CD9 shRNA cells, suggesting the implication of CD9 in their proper morphogenesis. In contrast, many dorsal ruffles were present (Fig. 3G, green asterisks; Supplementary Fig. S1).

**Figure 4:**
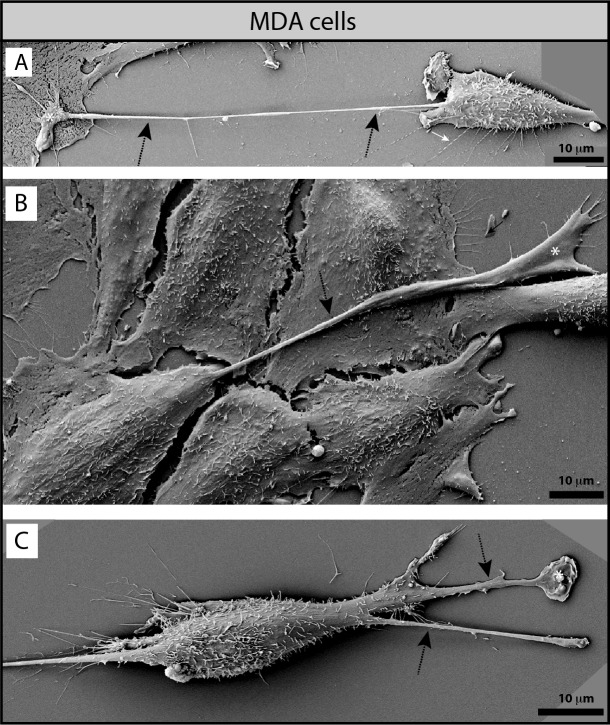
MDA cells display magnupodia **A**-**C**. MDA cells grown on poly-L-lysine-coated coverslips were analyzed by SEM. MDA cells develop long and thick magnupodia (dashed arrows) where a lamellipodium often emerges from their ending point (white asterisk). Scale bars are indicated.

Interestingly, MDA cells developed very long, and often thick (0.5-1.2 μm) PMPs (referred to as magnupodia), which extended for up to 100 micrometers away from the cell body and adhered either to the substratum or to another cell (Fig. 4A-C, dashed arrows). Frequently, a lamellipodium was found at their tip (Fig. 4A-C, white asterisks). Sometimes, the magnupodium seemed not to interact directly with the substratum with the exception of its ending lamellipodium (Fig. 4A). Generally, the MDA cells harboring a magnupodium had fibroblast-like structure (Fig. 4). Magnupodia were detected less often in MDA CD9 shRNA cells (Table 1), suggesting that CD9 is somehow involved in their formation and/or stabilization (see below for the localization of CD9 in these PMPs). Finally, both parental and CD9-deficient MDA cells exhibited numerous small and thin processes emerging from their dorsal part, which were able to link two neighboring cells or to establish a contact with the substratum (Fig. 3, Supplementary Fig. S1, white arrows; Table 1).

**Figure 5:**
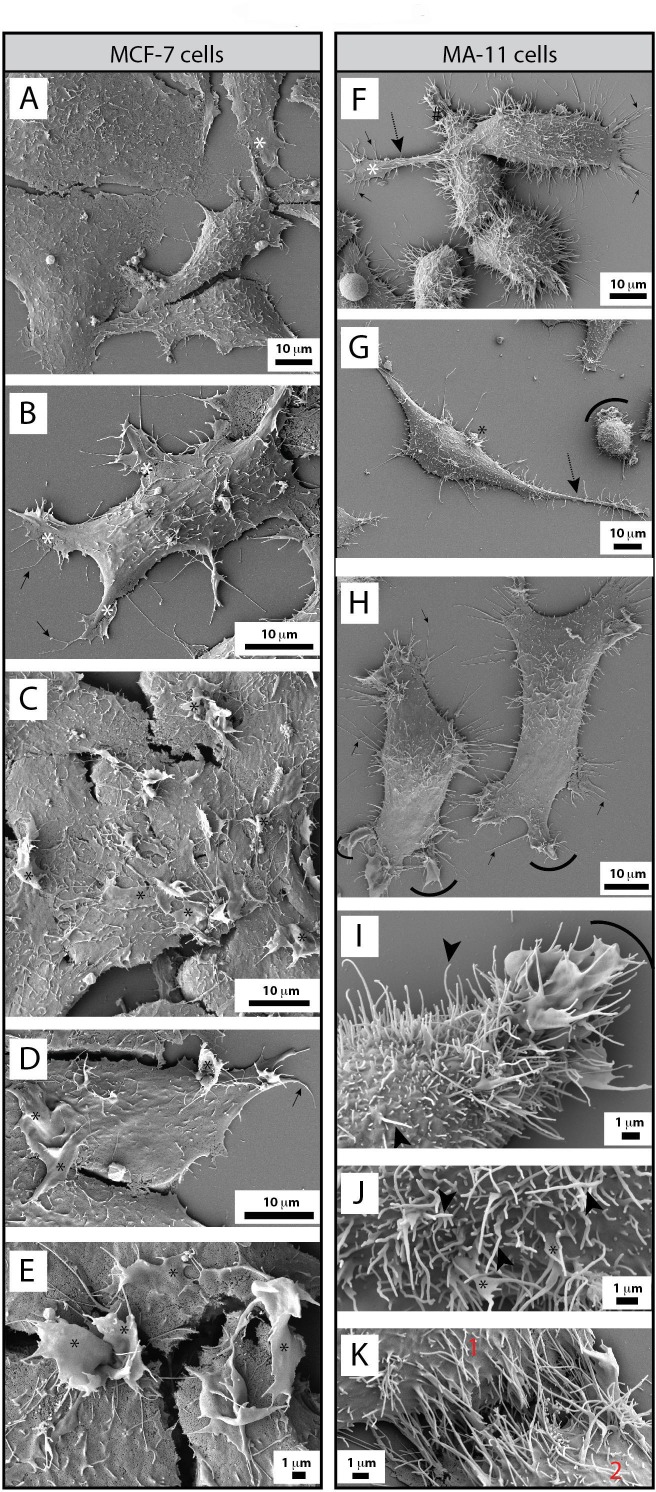
MCF-7 and MA-11 BCC lines are morphologically distinct to MDA cells **A**-**K**. MCF-7 (A-E) and MA-11 (F-K) cells grown on poly-L-lysine-coated coverslips were analyzed by SEM. MCF-7 cells developed various types of PMPs such as lamellipodia (A, B, white asterisks), filopodia (D, black arrows) and large dorsal ruffles (C-E, black asterisks), the latter structure being absent in MDA cells. In contrast, MA-11 cells are very often covered by long microvillus-like structures (F-K, black arrowheads). Rare magnupodia also contain them (F, G, dashed arrow). Although infrequent at the dorsal portion of the cells (G, black asterisk), membrane ruffles were observed at the cell periphery (G-I, arcs), which also contained filopodia (F, H, black arrows). Microvillus-like structures seem often to link two neighboring cells (K, 1 and 2). Broken membranes during the SEM preparation are indicated with #. Scale bars are indicated.

A similar analysis was performed on MCF-7 and MA-11 cells. MCF-7 cells were spread and created lamellipodia/filopodia as observed for MDA cells (Fig. 5A, B, white asterisks and black arrows, respectively). No magnupodium was observed in MCF-7 cells or very rarely with MA-11 cells (Fig. 5F, G, dashed arrows). Likewise, no or rare thin processes emerging from their dorsal part could be detected. In contrast, MCF-7 cells harbored large dorsal ruffles (Fig. 5B-E, black asterisks). MA-11 cells also produced lamellipodia/filopodia (Fig. 5F-H, white asterisks and black arrows, respectively) and developed peripheral ruffles similar to CD9-deficient MDA cells (Fig. 5F-H, arcs). Remarkably, MA-11 cells were often covered with numerous long microvillus-like structures (3.013 ± 0.948 μm by comparison to 1.069 ± 0.146 in MDA cells, n > 100; Fig. 5F-J, black arrowheads). The latter structures are particularly interesting since they seem to link adjacent cells (Fig. 5K).

**Table 1:** Distinctive morphological traits and common features of different breast cancer cell lines

	MDA	CD9shRNA (MDA)	MA-11	MCF-7
Shape and growing profile	Spindle, and sometimes growing above each other	Polygonal, cell cluster	Polygonal, cell cluster	Polygonal, cell cluster
Morphology at the cell border	Lamellipodia and filopodia		Lamellipodia and filopodia	Lamellipodia and filopodia
		Peripheral ruffles	Peripheral ruffles	
	magnupodium (length 73 ± 27 μm; thickness 0.5-1.2 μm) (n = 9)	Very rare magnupodia	Rare magnupodia	No magnupodium
Morphology at the cell surface	Microvillus-like structures (length 1.1 ± 0.1 μm; thickness 0.11 ± 0.01μm) (n = 96)	Small dorsal ruffles (height 1.1 ± 0.3μm; width 1.6 ± 0.3 μm) and/or altered microvillus-like structures (n = 30)	Long microvillus-like structures (length 3.0 ± 1 μm; thickness 0.14±0.02 μm) and thin ruffles (n = 138)	Small microvillus-like structures (length 1.1 ± 0.2 μm; thickness 0.11 ± 0.01μm) and ruffles (n = 31)
	Thin (0.08 ± 0.02 μm) and long (47 ± 12 μm) processes with small membranous bulges (n = 21)	Thin (0.08± 0.01 μm) and long (48 ± 18 μm) processes with small membranous bulges (n = 14)		
				Sparse and large dorsal ruffles (height 5.6 ± 1.6 μm; width 6.1 ± 1.7 μm) (n = 42)
Cell-cell contact	Lamellipodia that grow above the neighboring cells		Long microvillus-like structures and thin ruffles emerge from the cell border and make contacts with adjacent cells	Large dorsal ruffles are touching neighboring cells
	Thin and long processes emerging from the apex (or side) of cell are connecting adjacent cells	Thin and long processes emerging from the apex (or side) of cell are connecting adjacent cells		
	Frequent magnupodia that links cells over a long-distance			

The data are mean ± standard deviation of indicated number (n) of measurements

### CD9 is present in several types of BCC PMPs

**Figure 6:**
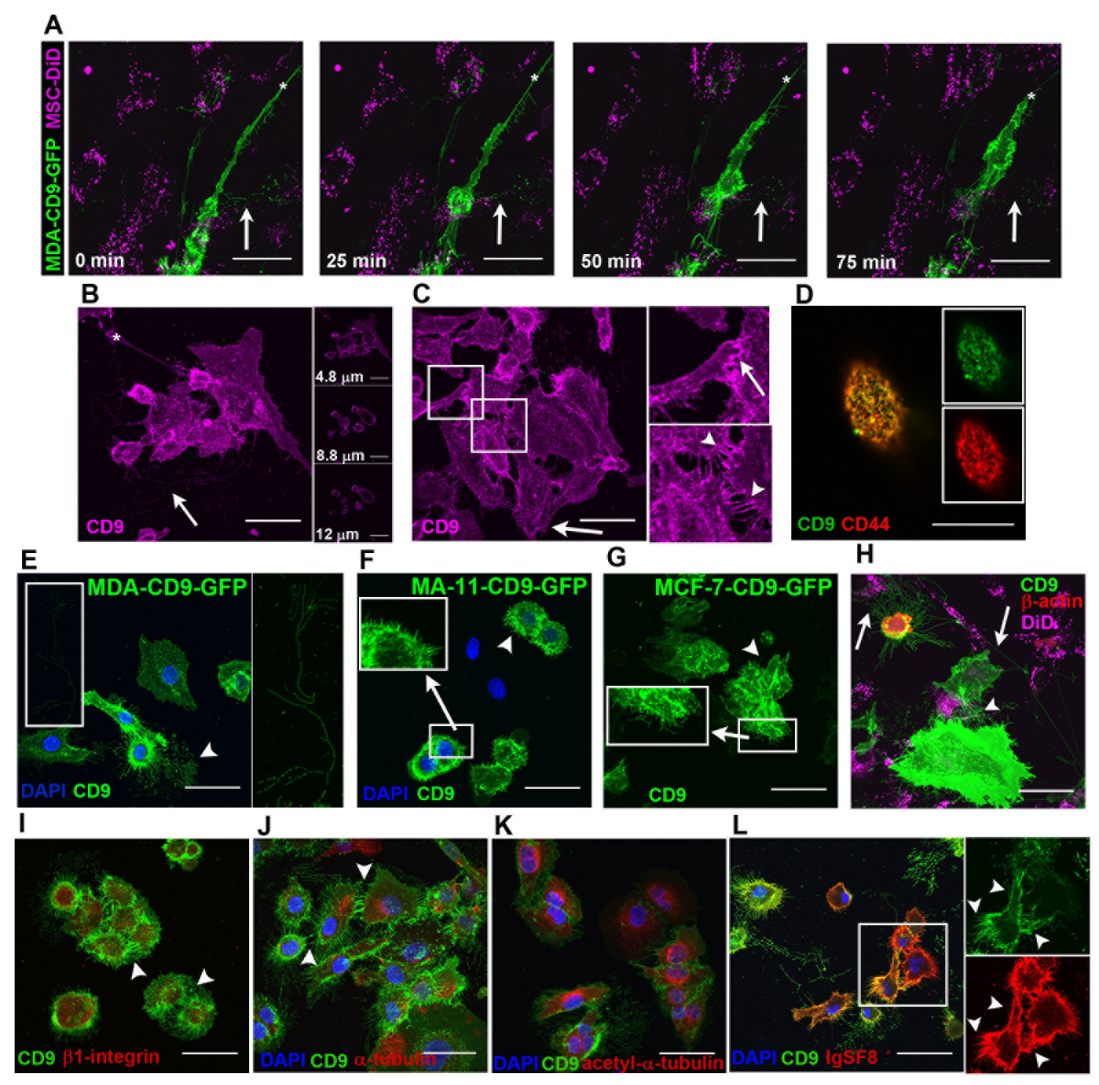
CD9^+^ PMPs on BCCs **A.** Confocal images were taken every 25 min of live CD9-GFP-transfected MDA cells cultured with DiD-labeled MSCs. Retraction of magnupodium (asterisk) occurs on a 1 h-time scale. Arrows, branched CD9^+^ filopodia detached from cells and degraded over time. **B**. **C**. Fixed, permeabilized MDA cells were stained with anti-CD9 Ab followed by Cy5-conjugated secondary Ab. Images are maximum intensity projections (MIPs) of confocal z-stacks. Representative z-optical sections taken at different heights from the coverslip shown to the right of the MIP. Arrow, branched CD9^+^ filopodia; white asterisk, lamellipodium at the tip of magnupodium (B). CD9^+^ PMPs such as a lamellipodium (arrow) at the tip of magnupodium and short filopodia (arrowheads) promote the intercellular interaction between two MDA cells. High power views of images of outlined areas are shown (C). **D**. CD9-GFP-transfected MDA cells stained with anti-CD44 Ab followed by Cy5-conjugated secondary Ab. Image is representative z-optical section of microvilli. Insets show GFP and Cy5 channels. **E**-**G**. CD9^+^ PMPs on MDA-CD9-GFP, MA-11-CD9-GFP, and MCF-7-CD9-GFP cells. Branched CD9^+^ filopodia (arrowhead) are emerging from MDA cells over long distance (see magnification of the outlined area) (E). Short CD9^+^ filopodia (arrowhead) are emerging from MA-11 (F) and MCF-7 (G). Note numerous CD9^+^ long microvilli in MA-11 cells and large dorsal ruffles (intense green areas) in MCF-7 cells (F, G, respectively, see insets where magnifications of indicated areas are shown). **H**. Live-cell image of CD9-GFP and β-actin-mCherry-transfected MDA cells co-cultured with DiD-labeled MSCs. CD9^+^ long and branched filopodia (arrows) and thin membrane processes (arrowhead) are either spreading over the long distance or link adjacent cells. **I**-**L**. CD9-GFP-transfected MDA cells were labeled with anti-β1 integrin (I), anti-α-tubulin (J), anti-acetylated-α-tubulin (K), or anti-IgSF8 (L) Abs followed by Cy5- or TRITC-conjugated secondary Ab. CD9^+^ filopodia are indicated with arrowheads. High power views of CD9^+^IgSF8^+^ PMPs of outlined areas are shown (L, GFP and TRITC channels). MIPs of confocal z-stacks (A-C, E-K). All scale bars, 50 μm, except for D, 25 μm.

TIRF and confocal live cell-imaging of MDA cells revealed an extensive array of CD9^+^ PMPs. CD9 was observed either by immunolabeling or upon the ectopic expression of CD9-GFP fusion protein. Both approaches gave similar data indicating that fusion protein is properly targeted to PMPs (Fig. 6A-E). CD9 is detected in (i) microvilli; (ii) branched web-like filopodia, which are morphologically similar to the described nanopodia that play a role in cell adhesion [27]; (iii) unbranched filopodia bundles, associated with directional movement (see below); (iv) short filopodia; (v) magnupodia; and (vi) lamellipodia (Fig. 6C, arrow). The different CD9^+^ PMPs were present either when MDA cells were cultured alone (Fig. 6B, C) or with DiD-labeled MSCs (Fig. 6A, H). As observed by SEM, MDA cells deployed a long magnupodium away from the cell body towards a neighboring one (Fig. 6B, white asterisk). Magnupodia tended to float in the medium and, with fluid motion, to extend downstream, whereas the cell remained adherent to the substratum (Fig. 6B). The morphology of magnupodia suggests that they may play sensory and mechanical roles during cell migration. During retraction, the extended materials often pooled into a bulb located on the pod (Fig. 6A). Both MDA and MDA-CD9-GFP cells contained CD9^+^ PMPs between them that could play a role in cell-cell adhesion (Fig. 6C, I, J, arrowheads).

We then evaluated the number of CD9^+^ PMPs emerging from MDA, MA-11 and MCF-7 cells transfected with CD9-GFP fusion plasmid by confocal microscopy (Fig. 6). In both MA-11 and MCF-7 cells, shorter and less frequent CD9-GFP^+^ PMPs than MDA cells were observed as suggested by SEM analyses. The calculated area of PMPs for MDA cells was 4200.0 +/− 732.1 μm^2^, 1583.3 +/− 215.0 μm^2^ for MA-11 cells, and 716.7 +/− 269.7 μm^2^ for MCF-7 cells (for technical details see Methods). The numbers of CD9-GFP^+^ PMPs is significantly higher in MDA cells by comparison to MA-11 (p = 0.02) and MCF-7 (p = 0.01) cells. MA-11 cells did have a greater frequency of CD9-GFP^+^ PMPs than MCF-7 cells (*p* = 0.047).

**Figure 7:**
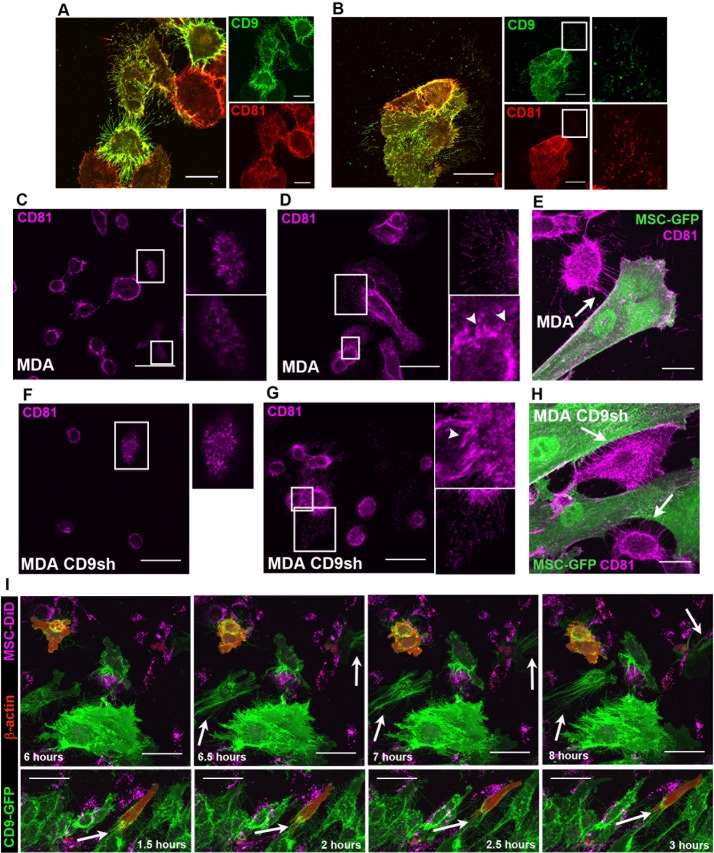
CD9^+^ PMPs contain CD81 and actin **A**. **B**. CD9-GFP-transfected MDA cells were fixed, permeabilized and labeled with anti-CD81 Ab followed by Cy5-conjugated secondary Ab. A co-localization of CD9 with CD81 was observed on PMPs. Short (A) and long (B) CD9^+^CD81^+^ filopodia and cell footprints (B, inset) are shown. Individual GFP and Cy5 channels are shown (right panels). Images are MIPs of z-stacks. **C**-**H**. MDA (C-E) or MDA-CD9shRNA (F-H) cells were plated alone (C, D, F, G) or with MSCs-GFP (E, H) prior to labeling with anti-CD81 Ab. Arrows indicate BCC-derived thin filopodia interacting with a MSC (E, H), while arrowheads point to short and thin processes connecting cells (D, G). Magnified images of outlined regions are shown (C, D, F, G). Images are MIPs (D, E, G, H) or representative optical sections of z-stacks (C, F). **I.** MDA-CD9-GFP-β-actin-mCherry cells cultured with DiD-labeled MSCs were imaged every 30 min for a 12 h period. Arrows indicate CD9^+^ filopodia approximately 180^o^ opposite to the direction of cell movement (top panels) or actin at the base of these filopodia (bottom panels). Images are MIPs of z-stacks. Scale bars are 50 μm, except for A, E, H, 25 μm.

Interestingly, CD9^+^ filopodia and thin PMPs were negative (or below the detectable level) for α-tubulin (acetylated and non-acetylated) and β1 integrin (Fig. 6I-K). IgSF8, a binding partner of CD9, was located along CD9^+^ filopodia (Fig. 6L). CD44, which is known to associate with CD9, was observed in CD9^+^ PMPs including microvilli (Fig. 6D). CD9 and CD44 showed a strong co-localization with a Pearson's co-localization coefficient of 0.87 +/− 0.02. Similarly, CD9 co-localized with CD81 on the plasma membrane and PMPs thereof (Pearson's R value 0.82 +/− 0.04) (Fig. 7A, B). A co-localization of CD9 and CD81 was also observed in filopodia and cell footprints (Fig. 7A, B, respectively), the latter being fragments of PMPs that remain attached to the substratum when cells are migrating further [28]. These footprints were degraded over time (Fig. 6A, white arrows). CD81 was also detected at the apex of parental MDA/MDA control shRNA and MDA-CD9 shRNA cells where microvillus-like structures and small dorsal ruffles are found, respectively, (Fig. 7C, F; Supplementary Fig. S2). Likewise, numerous thin membrane processes with small membranous bulges that establish a contact with the substratum (Fig. 7D, G) or with either neighboring MDA cells (Fig. 7D, G, Supplementary Fig. S2, arrowheads) or MSCs-GFP (Fig. 7E, H, Supplementary Fig. S2) were positive for CD81. Given the localization of CD81 and CD9 in various types of PMPs this alternative marker allows us to quantify the number of PMPs in CD9-deficient MDA cells. Fluorescence measurements of CD81^+^ PMPs were not significantly different between MDA (272.3 +/− 41.9), MDA CD9shRNA (372.6 +/− 41.9) and MDA control shRNA (354.1 +/− 26.5) cells, suggesting that the knockdown of CD9 did not reduce them with the notable exception of magnupodia (see above; Supplementary Fig. S2). Although the total expression level of CD81 was increased upon CD9 knockdown as observed by immunoblotting (Fig. 1F), the lack of intensified immunofluorescence signal in MDA CD9shRNA cells might be explained by its oligomerization or other protein-protein interactions where certain CD81 epitopes will be masked. Neither the morphology of MDA cells nor the number of CD9^+^ PMPs derived therefrom were affected when they were transduced with control shRNA (Supplementary Fig. S2).

MDA-CD9-GFP cells were transfected with a β-actin-mCherry fusion plasmid to determine the relationship between the dynamics of filopodia and cell movement, and the presence of actin in CD9^+^ PMPs. When MDA-CD9-GFP-β-actin-mCherry cells were co-cultured with DiD-labeled MSCs, unbranched filopodia bundles containing CD9 appeared at a 163^o^ +/− 14^o^ (s.d.) angle from the direction of MDA cell movement (Fig. 7I, top panel). The average ratio of unbranched filopodia expansion/distance traveled by the cell was 0.82 +/− 0.089 (s.d.). This indicated that as a MDA cell migrated, the distance traveled was almost equal to the length of the growing filopodia. The actin was located solely at the base of the filopodia bundles during cell migration, and consequently did not extend through the length of the CD9^+^ PMPs (Fig. 7I, bottom panel). Together, these observations suggest that trailing filopodia could influence the speed and direction of the cancer cell migration. Thus, they might guide the invasion cells in a 3-dimensional (3D) microenvironment.

**Figure 8:**
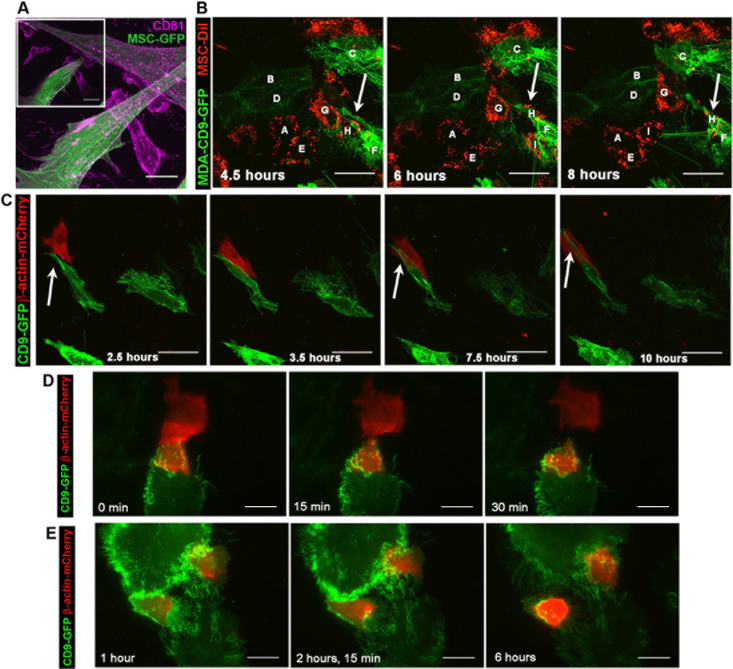
The invasion of MSCs by MDA cells involves CD9^+^ lamellipodia **A.** MDA cells were cultured with GFP-transduced MSCs prior to their staining with anti-CD81 Ab. Inset shows a representative z-optical section of MIP of z-stack. **B.** Live-cell confocal images of CD9-GFP-transfected MDA cells (*B*, *C*, *D*, *F*) co-cultured with DiI-labeled MSCs (*A*, *E*, *G*, *H*, *I*) were taken every 30 min for at least 8 h. A lamellipodium-driven invasion of MDA-CD9-GFP cell (cell *F*) into DiI-labeled MSC (cell *H*). **C**. Live-cell TIRF images of MDA-CD9-GFP cells cultured with β-actin-mCherry-transfected MSCs were taken every 30 min for at least 10 h. A CD9^+^ lamellipodium (arrow) emerging from a MDA cell interacts with MSC prior to invasion. **D**. **E**. Live-cell TIRF images of MDA-CD9-GFP cells co-cultured with β-actin-mCherry-transfected MSCs were acquired every 15 min. The indicated time points are shown. Note a large piece of MSC phagocytosed by MDA cell (D). Two distinct MDA-CD9-GFP cells contain fragments of β-actin-mCherry^+^ MSC (E). Scale bars, A, C-E, 25 μm; B, 50 μm.

Magnupodia with lamellipodia at their leading edge containing both CD9 and CD81 were observed in MDA cells as well as MDA-CD9-GFP cells (Fig. 8A, C, Supplementary Fig. S2D). These lamellipodia were involved in MDA cell invasion into MSCs, as shown in co-cultures of MDA/MDA-CD9-GFP cells with MSC-GFP/MSCs, the latter ones being either labeled with DiI or transiently transfected with β-actin-mCherry (Fig. 8A-C; Supplementary Video S3). Live-cell TIRF imaging of co-cultures of MDA-CD9-GFP with MSC-β-actin-mCherry cells showed the formation of a CD9^+^ magnupodium extending from an MDA cell (Fig. 8C), similar to those observed by SEM (Fig. 4). Upon its initial contact with the MSC, a cell invasion occurred. Given that the CD9 deficiency dramatically reduced the number of MSC invasions and no magnupodia was observed in MDA CD9shRNA cells, these observations suggest that CD9-dependent magnupodia is the main PMPs of MDA cells involved in the invasion of MSCs.

Interestingly, TIRF imaging revealed a progressive transfer of materials from MSCs-β-actin-mCherry to MDA-CD9-GFP cells. Thus we could observe a large portion of an MSC being taken up by an MDA-CD9-GFP cell (Fig. 8D, E and Supplementary Video S4). Although such phenomenon is rare (~1-2 events/50,000 cells) under our experimental setting, it is nonetheless observable.

### CD9 is required for generation of heterotypic hybrids

Next, we investigated the formation of BCC/MSC hybrids by culturing them together for 6 days. Interestingly, cellular invasion was associated with spontaneous fusion of MDA or MA-11 cells with MSCs, which amounted to 0.68 and 0.12% according to initial number of plated BCCs, respectively (Table 2). The pre-incubation with two different CD9 blocking Abs caused a decrease of fusion between MDA cells and MSCs. The knockdown of CD9 in both cell lines totally abrogated the heterologous fusion. No fusion events were observed between the less aggressive MCF-7 cells or HuMECs with MSCs (Table 2).

**Figure 9:**
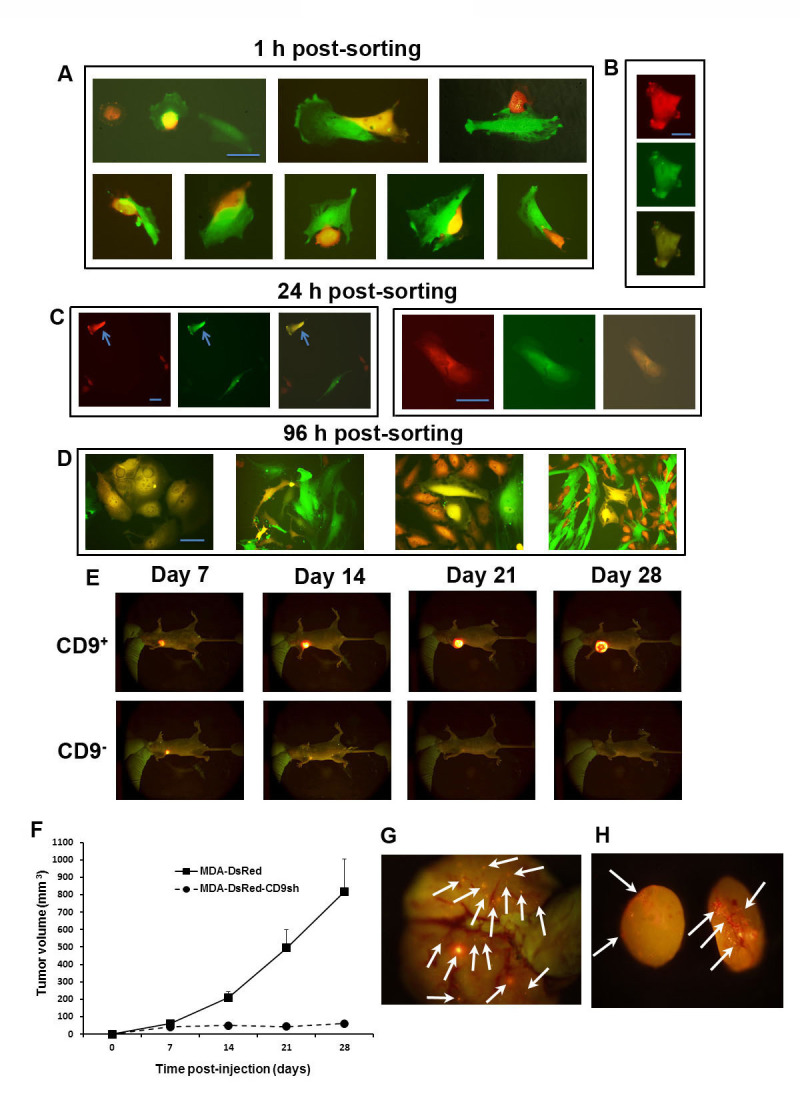
CD9-dependent fusogenicity, tumorigenicity and metastatic capacity of MDA cells **A**-**D**. The great majority of dual-fluorescent MDA-MSC hybrids from 5-days co-cultures of DsRed-transduced MDA and GFP-transduced MSCs represents a transient stage of invasion. Representative MDA-MSC hybrid observed 1 h (A, B) or 24 h (C, arrow) post-sorting. Groups of proliferating hybrids observed 96 h post-sorting (D). Scale bars, 30 μm; **E**. Fluorescence imaging over time of two representative tumors in the mammary fat pad, upon injection of 6 × 10^6^ MDA-DsRed (upper panels) and MDA-DsRed-CD9shRNA (lower panels) cells in *nu/nu* mice. **F**. Analysis of primary tumor growth by live-animal fluorescence imaging. Tumor volumes were evaluated weekly over 1 month period and analyzed by two-way ANOVA (p<0.01). Error bars, s.e.m. from the mean volume of ten xenografts per group. **G**. **H**. Representative fluorescent images of lung (G) and axillary lymph node (H) metastases in *nu/nu* mice six weeks after injection of MDA-DsRed cells.

Clearly, most invasion events did not result in heterologous fusion. To investigate the relationship between invasion and cell fusion, we sorted and re-plated dual (red-green) fluorescent cells originated from mixed BCC/MSC co-cultures. When analyzed within 60 min from sorting, cells showed different morphological stages of invasion and presence of dual fluorescent cells (about 30%) (Fig. 9A, B). However, 24 hours later, about 1% of the double-positive cells were present (Fig. 9C). As shown in Table 2 and Fig. 9D, the latter fraction of cells included viable hybrids, i.e. able to divide and form dual fluorescent progeny with mesenchymal morphology, as previously described [11]. Disappearance of dual fluorescent cells could not be attributed to selective cell death of the hybrids, since trypan blue dye exclusion revealed that was over 99% of cell viability. Indeed, the majority of sorted dual-color cells was confirmed not to be stable hybrids, four days after sorting, by a single broad DNA peak with a DNA index of 1.2 including both diploid MSCs (DNA index 1.0) and MDA cells (1.25). These data suggest that the double-fluorescent cells observed by flow cytometry represented for the most part transient stages of invasion. We observed occasional occurrence of entosis, with presence of Ds-Red in the cytoplasm of MSCs, evidenced by yellow fluorescence (Fig. 1G, mix of red and green).

**Table 2:** Effect of anti-CD9 antibodies and CD9 knockdown on formation of breast cancer/MSC hybrids

MDA/MSC	% hybrids ± S.D.^1^	P value
Control	0.68 ± 0.16	
Anti-CD9 (MEM-61)	0.15 ± 0.10	0.007
Anti-CD9 (M-L 13)	0.184 ± 0.16	0.016
Anti-CD9 (P1/33/2)	0.5 ± 0	0.11
CD9 knockdown	0	0.0018
**MA-11/MSC**		
Control	0.12 ± 0.05	
CD9 knockdown	0	0.022
**MCF-7/MSC**		
Control	0	
CD9 knockdown	0	
**HuMEC/MSC**		
Control	0	

1 2000 BCCs and 8000 MSCs were plated together (24-well plate) and dual fluorescence of heterotypic hybrids was counted after 6 days of co-cultures. The percentage of hybrids was calculated relative to the initial number of BCCs. Data are the mean of five independent experiments. Anti-CD9 Ab clone is indicated in bracket.

### CD9 knockdown inhibits tumor growth and metastases in immune-deficient hosts

To evaluate the effects of CD9 knockdown on MDA cells *in vivo*, we compared primary tumor growth and metastasis after implantation of either MDA-Ds-Red or MDA-Ds-Red-CD9sh cells into the mammary fat pad of *nu/nu* mice. The tumor progression was monitored weekly by orthotopic tumor fluorescence. The CD9-deficient cells could initiate the growth of tumor until 7^th^ day post-injection in agreement with *in vitro* data (Fig. 1E), however, the cancer progression was afterward stopped and in several cases a complete regression was observed (Fig. 9E, F). In mice implanted with MDA-Ds-Red cells, macroscopic fluorescent axillary lymph node, lung and brain metastases were detected in 70, 60 and 40% of the animals at autopsy, respectively, by comparison these numbers drastically dropped to 30, 40 and 0% of mice implanted with MDA-Ds-Red-CD9sh cells. Representative fluorescent images of lung and lymph node metastases of nu/nu mice implanted with MDA-DsRed cells are shown in Fig. 9G, H.

## DISCUSSION

Breast cancer progression into overt metastatic phenotype requires migration of tumor cells through extracellular matrix and across host cell layers. In the present study, we characterized CD9^+^ PMPs of BCCs as pro-invasive and fusogenic, found a direct correlation between the extent of CD9^+^ PMPs and BCC invasiveness and established CD9 as a molecule required for the correct formation of certain types of PMPs, notably the long magnupodium.

**Figure 10:**
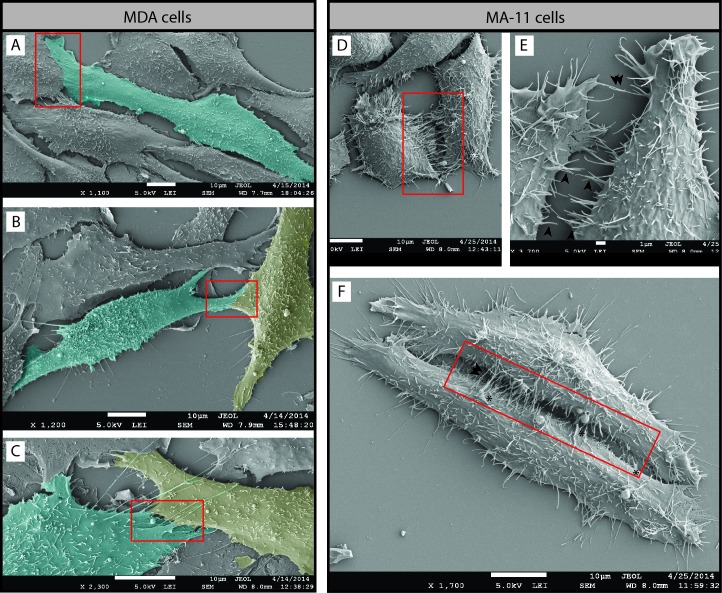
Two distinct potential mechanisms involved in cancer cell invasion and fusion **A**-**F.** MDA (A-C) and MA-11 (D-F) cells grown on poly-L-lysine-coated coverslips were analyzed by SEM. In MDA cells, a lamellipodium emerging from one or two cells simultaneously seems to link them closely (red box). Note that in certain cases it is highly difficult to delimitate the cell border suggesting membrane fusion (B, C). Cells were pseudocolored in cyan or yellow in order to help their visualization. In MA-11 cells, microvillus-like structures (E, black arrowhead) and thin membrane ruffles (F, asterisk) emerging from cell borders (red box) might play a role in cell-cell contacts, invasion and eventually cell fusion. Note that rare MA-11 cells with an elongated morphology (F) can generate a microvilli zipper between their entire length. Scale bars are indicated.

In MDA cells, migration, invasiveness and subsequent heterologous fusion were achieved by CD9-dependent lamellipodia including those found at the tip of magnupodia (see Figs 4, 10), which in *in vivo* 3D-microenvironment could appear as invadopodia. The absence of CD9 impaired their formation and/or destabilized them, which resulted in numerous membrane ruffles at the edge of MDA CD9shRNA cells, and hence the lack of firm adhesion therein. Interestingly, while CD9 knockdown and blocking Abs severely hindered invasion and cell fusion, the overall adhesion properties of MDA CD9shRNA cells to MSCs were nonetheless preserved or seemingly increased. These data are in agreement with earlier results showing that the lack of fusion in *Cd9* null eggs was not accompanied by loss of sperm–egg adhesion [24, 29], but rather by an increase in sperm binding sites [30]. Such observation suggests that CD9 deficiency lead to a compensatory mechanism responsible for the maintenance of cellular adhesion. The increased expression of CD9-interacting partner CD81 observed in CD9-deficient cells might explain it. CD81 was constantly associated with CD9^+^ PMPs. We are currently using atomic force microscopy-based single-cell force spectroscopy to quantify the adhesive BCC-MSC interaction(s) and decipher further these issues. Nonetheless, the up regulation of CD81 did not compensate the MSC invasion *in vitro* or cancer progression and metastasis capacity of CD9-deficient MDA cells *in vivo* in agreement with previous studies (see below; reviewed in Ref [12]).

It is worth to mention that thin CD9^+^ PMPs, which often interact with those emerging from neighboring cells (Fig. 3, white arrows), are reminiscent to long and thin nanopodia recently described in endothelial cells [27]. Like those described here, they contain a tetraspanin protein, namely transmembrane-4-L-six-family (TM4SF1). The function of such TM4SF1/CD9^+^ membrane projections is not yet clear but they might play a role in intercellular communication or sensing the microenvironment. In addition, the implication of CD9^+^ filopodia in keratinocyte wound healing migration or lymphocyte trans-endothelial migration has been previously reported [28, 31, 32]. Obviously, further efforts are urged to determine their implication in cancer progression.

Magnupodia and thin PMPs are rarely observed in MA-11 cells, and their invasiveness and fusogenicity might be related to the long microvillus-like structures (~3 μm in length) that originate from the cell border, and collectively form a “microvilli zipper” between adjacent BCCs (Fig. 10). The formation of CD9-dependent microvilli zippers is not unique to cancer cells since similar structures have been demonstrated to regulate virus-induced cell fusion [33]. Thus, CD9^+^ microvillus-like structures could act as a platform to concentrate adhesion and/or fusion proteins such as CD44. Is CD9 directly involved in the microvillar architecture? A hint of its direct structural role was already provided in *Cd9* null oocytes, where numerous alterations (length, thickness and density) in microvillus-like structures were noted [32, 34]. Similarly, we observed that CD9 knockdown causes alterations of microvillus-like structures at the dorsal part of MDA cells, which resulted in the appearance of tiny dorsal ruffles. CD9 proteins together with the tetraspanin web could provide the appropriate shape of PMPs including a low radius of curvature necessary for membrane fusion [32, 35]. Finally, we could not exclude that dorsal microvillus-like structures of MDA cells participate as well in cellular interaction/fusion *in vivo* given that they might be in direct contact with adjacent cells in a native microenvironment in contrast to 2D cell cultures. Besides the components of PMPs (i.e. lamellipodia/magnupodia in MDA cells and microvillus-like structures in MA-11 cells), other actors may be involved in bridging adjacent membranous structures *in vivo*, such as soluble factors and/or microvesicles [36]. Interestingly, CD9 is a major constituent of exosomes released by MDA cells (Green et al., unpublished data). Since inflammation is often associated with tumor microenvironment, adhesion-promoting factors released by hematopoietic cells could also play a role [37].

The impairment of BCCs invasion into MSCs, caused either by CD9 deficiency or Ab interference, led to decrease of heterotypic cell-cell fusion – potential source of genomic instability, aneuploidy and cancer heterogeneity – hallmarks of cancer progression. A direct relation between invasiveness and fusogenicity is nonetheless not surprising, in view of previous findings showing that in high-density homotypic cultures, MDA cells were 11- and 7.2-fold more fusogenic and capable of invading Matrigel matrix, respectively, than MA-11 cells [38]. Although only a minority of invasion events resulted in formation of hybrid cells (e.g., MDA/MA-11–MSC), our previous study demonstrated that after 5 days of drug selection 80% of cells in mixed cultures were hybrids, capable of forming metastatic tumors in immune-deficient hosts, suggesting that the current phenomenon is biologically pertinent [11]. In addition, we occasionally observed in our co-cultures a phenomenon entosis, which is implicated in cancer aneuploidy and progression [39], our adherent BCCs and MSCs were not favorable to its occurrence since the entosis mainly happens in suspension cultures [39-41].

It might not be more a coincidence that MCF-7 a much less aggressive and poorly metastatic BCC line [42], displays less CD9^+^ PMPs than MDA cells and has poor invasive and fusogenic capacities. These observations strongly suggest that local cellular interactions in the microenvironment, presumably mediated by CD9^+^ PMPs, play an important role in the breast cancer metastatic phenotype. While MDA cells are triple negative (estrogen receptor (ER), progesterone receptor (PR), Her-2), and belong to Basal-like subtype of breast carcinomas, MCF-7 cells are ER^+^/PR^+^/Her-2^−^ and fell within Luminal-A subtype [43]. Tumors of Basal-like subtype are aggressive, poorly differentiated and often occur on younger patients with a poor prognosis by comparison to those of Luminal-A subtype, which are more differentiated and observed in older patients with the best prognosis contrasted to other subtypes [43]. Thus, the presence and/or absence of certain types of PMPs (e.g., magnupodium, long microvilli, membrane ruffles) can contribute, in addition to the molecular signature, to the classification of breast carcinomas.

The requirement of CD9 for orthotopic tumor growth suggests that membrane extension-driven cell communication and/or reduced cell adhesiveness to stromal components are responsible for the absence of local tumor growth. We should also keep in mind that CD9-deficiency leads to decreased expression of ADAM10, a metalloproteinase that could play a role in breast cancer progression [44]. Nonetheless, the formation of metastases, although reduced, was not fully abrogated by CD9 knockdown. This may reflect the capacity of BCCs of intra/extravasating *in vivo* through tight endothelial junctions rather than trans-cellular route and/or to decreased adhesiveness caused by CD9 deficiency that could facilitate metastatic dissemination. Recent findings that the CD9/CD81 tetraspanin complex regulates α3β1 integrin-dependent tumor cell behavior [45] and that integrin α3β1 promotes breast carcinoma metastases [46] suggests that the anti-metastatic effect observed upon CD9 knockdown could depend on the CD9 regulation of α3β1-integrin. However, we did not detect association between CD9 and β1-integrin in MDA cells.

Our findings are intriguing in the context of the “push and pull” role of tetraspanins in metastases [12]: while an inverse correlation between CD9 expression and metastatic potential was observed in some studies [12, 47], a pro-metastatic role of CD9 was observed in others [48-52]. Interestingly, while melanoma cells had reduced CD9 expression relative to normal melanocytes, forced increase in CD9 expression stimulated their invasiveness [53]. Similarly, CD9 was down regulated in primary sites of cervical cancers, but re-expressed at sites of trans-endothelial invasion to promote malignant expansion at metastatic sites [54]. Collectively, our data indicate that CD9 is implicated in BCC invasiveness and tumor growth by mechanisms that involve specific and dynamics protruding structures of BCC plasma membrane. Investigation of CD9-targeted breast cancer therapeutic strategies is warranted.

## METHODS

### Statistical Analysis

Error bars in graphical data represent means ± s.e.m., unless stated otherwise. Mouse experiments were performed in duplicate, using at least 5 mice per treatment group. To determine how many mice were required for the data to be statistically significant, we used a Fisher's Exact test at 80% power and 95% confidence ("http://calculators.stat.ucla.edu/powercalc/binomial/fishers/index.php). For tumor growth analyses, we performed two-way ANOVA statistical analysis. All *in vitro* experiments were performed at least in triplicate. Statistical significance was determined using a two-tailed Student's t-test, and p-values of *p* < 0.05 were considered statistically significant by ANOVA. For total internal reflection fluorescence (TIRF) analysis, F-tests were performed to compare variance between groups. Those that were determined to have equal variance were compared using an unpaired Student's t-test, and those that had unequal variances were compared using a Welch's t-test. The first BCC line studied was evaluated using a two-tailed Student's t-test, and subsequent BCC lines were evaluated using a one-tailed Student's t-test.

The Manual Tracking Plugin of Fiji was used to track MDA-CD9-GFP cell movement as well as the position of the CD9^+^ unbranched bundles. The angle of cell at the base of the extension compared to the tip of the extension was measured for the duration of cell movement using Fiji. Averages of at least 3 independent experiments are reported +/– s.d. The same Manual Tracking Plugin was used to measure the initial length of the CD9^+^ PMPs, the distance traveled by the cell, and the final length of the PMPs (measured using the last time frame of cell migration). The growth of the PMPs, i.e. difference between final and initial extension length, was divided by the distance traveled by the cell in order to calculate the ratio of unbranched filopodia growth: distance traveled by the cell. Averages of at least 3 independent experiments are reported +/− s.e.m.

### Cell Lines

Human MDA (i.e. MDA-MB-231) and MCF-7 BCCs were obtained from the American Type Culture Collection. The human MA-11 BCC line, which was established from bone marrow micrometastases of a breast cancer patient, was obtained from Dr. Fodstad (Norwegian Radium Hospital) [55, 56]. Human MSCs were isolated from 1–4 ml bone marrow aspirates taken from the iliac crest of normal adult donors after informed consent, as described [57]. They were obtained from Dr. Prockop (Texas A&M) and prepared under a protocol approved by the Texas A&M Institutional Review Board. MSCs were cultured in complete culture medium [α-minimal essential medium (Gibco, Carlsbad, CA), 17% fetal bovine serum (lot selected for rapid growth of MSC; Atlanta Biologicals, Inc., Norcross, GA), 100 units/ml penicillin, 100 μg/ml streptomycin, and 2 mM L-glutamine] as described [11]. Their multipotency was regularly monitored by their differentiation into adipocytes, osteoblasts and chondrocytes. All cell lines were kept in culture for less than 3 months. They were tested for mycoplasma contamination and authenticated by morphology.

### Antibodies

The following primary Abs were employed throughout this study: anti-CD9 clone P1/33/2 (Santa Cruz, Dallas, TX), anti-CD9 clone MEM-61 (Abcam, San Francisco, CA), anti-CD9 clone M-L13 (BD Biosciences, San Jose, CA), anti-CD9 clone HI9a (BioLegend, San Diego, CA), anti-CD81 (cat. #349502, BioLegend), anti-CD63 (cat. #sc-15363, Santa Cruz Biotechnology, Dallas, TX), anti-ADAM-10 (cat. #sc-25578, Santa Cruz Biotechnology), anti-β-actin (cat. #sc-47778, Santa Cruz Biotechnology), anti-IgSF8 (cat. #103561, Santa Cruz Biotechnology), anti-β1 integrin (cat #4706S, Cell Signaling Technology, Danvers, MA), anti-α-tubulin (cat. #sc-8035, Santa Cruz Biotechnology), anti-acetylated-α-tubulin (cat. #T7451, Sigma), anti-CD44 (cat. #MA5-13890, ThermoScientific, Waltham, MA). The following secondary Abs were employed: TRITC-conjugated anti-rabbit IgG (Jackson ImmunoResearch), TRITC-conjugated anti-goat IgG (Sigma), and Cy5-conjugated anti-mouse IgG (Jackson ImmunoResearch). For inhibition experiments, sodium azide was removed by desalting through Sephadex G-25 spin columns.

### Labeling of MSCs with Membrane Dyes

MSCs were enzymatically detached, centrifuged at 200 *x g* for 5 min, and resuspended in serum-free media. Cells were labeled for 10-20 min at 37^o^C by the addition of DiI (or DiD; Invitrogen, Grand Island, NY) at a final concentration of 5 μM. Cells were washed twice in complete medium, and plated in a 35-mm dish containing 0.17 mm coverslip. Labeled MSCs were co-cultured with MDA-CD9-GFP cells (ratio 1:1; 5 × 10^4^ cells) overnight prior to the beginning of the experiment.

### Retroviral and Lentiviral Vectors

The EGFP and DsRed-expressing retroviral vectors used are based on pSF91 (GenBank accession no. AJ224005) with the 3′ LTR of spleen focus-forming virus and the leader of the murine embryonic stem cell virus [58, 59]. To generate retroviral producers, the Phoenix-gp packaging cell line was transfected with pSF91/eGFP or pSF91/DsRed, and a plasmid expressing the vesicular stomatitis virus glycoprotein. Culture supernatants containing viral particles were collected at 24-48 h after transfection, passed through 0.22-μm Millex GP filters (Millipore Co., Bedford, MA) and stored at –80^o^C.

To inhibit the expression of CD9, shRNA lentiviral particles (Santa Cruz Biotechnologies, cat. #sc-35032-V) were employed. They consist of a pool of transduction-ready lentiviral vectors containing 3 target-specific constructs encoding 19-25 nucleotides (plus hairpin) shRNA designed to knockdown *CD9* gene expression. shRNA lentiviral particles containing an shRNA construct encoding a scrambled sequence (cat. #sc-108080) were employed as negative control.

### Viral Transduction

For transduction of MSCs and BCCs, retroviral supernatants or transduction-ready lentiviral particles were preloaded onto recombinant fibronectin (Retronectin, Takara Shuzo, Japan)-coated plates and centrifuged at 950 *x g* for 30 min at 4^o^C. The operation was repeated a second time with fresh supernatant. The supernatant was then removed and plates washed with PBS before addition of cells. After transduction, stable cell lines were selected by introducing 2 μg/ml puromycin in the culture medium.

### Transfection

MDA, MA-11 and MCF-7 cells were transfected by electroporation with 10 μg PrecisionShuttle mammalian plasmid encoding CD9 with GFP tag at its C-terminus under the control of the cytomegalovirus promoter (PS100010 vector; OriGene Tech., Rockville, MD) using a Gene Pulser X-cell electroporator according to the supplier's instructions (Bio-Rad, Hercules, CA). Cells expressing the neomycin resistance gene were selected by introducing 400 μg/ml of G418 (Life Technologies) into culture medium. At least one week before the experiments, G418 was omitted. Under these conditions, >99% of neomycin-resistant cells expressed the recombinant CD9-GFP fusion protein. MDA-CD9-GFP cells and MSCs were transiently transfected with pLVX-mCherry-Actin vector (0.8 μg DNA, Clontech, Mountain View, CA) using Lipofectamine 2000 (Invitrogen) in a 1:3 ratio of DNA:lipofectamine. Cells were plated in a 35-mm dish with 0.17 mm coverslip on the bottom and incubated for at least 16 h prior their use.

### Cell Sorting

Fluorescent cells were sorted using an SH800 cell sorter (Sony Global, Champaign, IL). To exclude doublets from the sorted sub-population of dual fluorescence hybrids, gating was performed sequentially on the basis of forward and back light scatter first, and then on the basis of forward scatter height vs. width.

### Deep RNA Sequencing (RNA-seq)

The RNA-seq-based mRNA profiling was performed by Ocean Ridge Biosciences (Palm Beach Gardens, FL) as previously described [11]. To normalize the data, the raw nucleotides mapped for each gene X sample were converted to reads per 100,000 total reads. Missing data points, where no reads were mapped for a specific sample X gene, were replaced with a value of 0.08 reads/100,000 total reads.

### Immunoblotting

Detergent cell lysates were prepared using lysing buffer (1% Triton X-100, 100 mM NaCl, 50 mM Tris-HCl, pH 7.5) supplemented with a protease inhibitor cocktail (Calbiochem, Billerica, MA). The lysates were incubated on ice for 30 min, centrifuged at 20,000 *x g* for 5 min, and Laemmli sample buffer (reducing condition) was added to the supernatants. Proteins were separated by a 4-12% Bis-Tris precast gel (Invitrogen) along with a pre-stained protein molecular weight ladder (Genetex, Irvine, CA), and transferred to a nitrocellulose membrane (Thermo, Waltham, MA). Membranes were incubated for 40 min in a blocking buffer (PBS + 1% BSA) at room temperature, and then probed with a given primary Ab overnight at 4^o^C. After 3 washing steps for 10 min each with PBS + 0.1% Tween 20, membranes were incubated with an IRdye secondary Ab (Licor, Lincoln, NE) for 30 min at room temperature. Finally, membranes were washed 3 times (10 min each) in PBS + 0.1% Tween 20, rinsed in ddH_2_O and antigen-Ab complexes were visualized using an Odyssey CLx system (Licor).

### Total Internal Reflection Fluorescence Microscopy

MSCs-GFP and BCCs were co-plated (6 × 10^4^ cells each) into 35-mm microscopy dishes containing coverslips (0.17 mm in diameter) (MatTek, Ashland, MA), and incubated overnight at 37^o^C prior the beginning of the experiment. Nikon Eclipse Ti inverted microscope equipped with a Nikon TI-TIRF Illuminator unit (Melville, NY) and a 60X Apo-TIRF oil-immersion objective (NA 1.49) was used to capture images. Cells were kept in a humid, live-cell chamber set at 37^o^C and 5% CO_2_ throughout the experiment. A 40 mW Argon ion laser tuned to 488 nm and a solid-state 561 nm laser were used to excite GFP and DsRed or β-actin-mCherry, respectively. Images were recorded using a Nikon Digital Sight DS-U3 CCD camera and processed using Nikon NIS Elements AR 3.22 software and Fiji. When indicated, 3 μg/μl anti-CD9 Ab (Abcam clone MEM-61) was added to the dish. Since the TIRF microscope excites fluorophores only ~100 nm from the coverslip, overlapping green and red fluorescence signified entry of the BCC into the MSC rather than cell stacking. Entries of BCCs were considered invasions if ≥50% of the cell was inside of, or passed through, a MSC.

### Confocal Microscopy

Cells (1 × 10^6^) of the indicated cell lines were plated into 35-mm microscopy dishes containing 0.17 mm coverslips on the bottom (MatTek) and incubated overnight at 37^o^C. Cells were washed with PBS, fixed in 4% paraformaldehyde (PFA) and permeabilized with 0.2% Tween 20 in PBS for 15 min at room temperature. Afterward, they were labeled with 25 μg/ml anti-CD9 Ab (clone HI9a, BioLegend), 2 μg/ml anti-β1 integrin Ab, 2 μg/ml anti-tubulin Ab, 10 μg/ml anti-acetylated tubulin Ab, 10 μg /ml anti-CD81 Ab, 4 μg/ml anti-CD44 Ab, or 4 μg/ml anti-IgSF8 Ab diluted in permeabilization buffer for 1 h at room temperature followed by TRITC-conjugated anti-rabbit (1:50) or anti-goat (1:100) IgG or 1:50 dilution Cy5-conjugated anti-mouse secondary Ab (Jackson ImmunoResearch or Sigma) for 30 min at room temperature. Cells stained with fluorescent secondary Ab alone were employed as a control. Cells were washed in PBS and, where indicated, stained with 1 μg/ml DAPI (MP Biomedicals, Santa Ana, CA) prior to imaging. Cells stained with TRITC- or Cy5-conjugated anti-mouse secondary Ab alone were used as a control.

Live cell imaging was performed in an incubation chamber at 37^o^C under 5% CO_2_. Cells were imaged using confocal laser-scanning microscopy on a Nikon A1R+ using a galvano or resonant scanner and a 60X Apo-TIRF oil-immersion objective with a numerical aperture of 1.49 at either 512 × 512 or 1024 × 1024 pixel resolution. 405 nm, 488 nm, 561 nm, and 638 nm solid-state lasers were used to excite DAPI, GFP, TRITC, and Cy5, respectively. DAPI, GFP, TRITC, and Cy5 fluorescence emissions were collected using 425-475 nm, 500-550 nm, 570-620, and 662-737 nm longpass filters, respectively. GFP and TRITC emissions were collected using higher-sensitivity GaAsP detectors on the A1R+ microscope. Images were recorded using NIS Elements software (Nikon) Raw images were processed using Fiji [60].

### Co-localization Analysis

MDA-CD9-GFP cells plated in 35-mm dishes with 0.17 mm coverslips on the bottom were fixed with 4% PFA for 20 min at 4^o^C. The cells were permeabilized with 0.2% Tween 20 in PBS for 15-30 min at RT, then stained with 4 μg/ml anti-CD44 or anti-CD81 Abs for 1 h. The cells were washed and stained with a 1:50 dilution of Cy5-conjugated anti-mouse secondary Ab (Jackson ImmunoResearch) for 30 min. Cells stained with Cy5-conjugated anti-mouse secondary Ab alone were employed as a control. The cells were washed with PBS prior to viewing on a Nikon A1R+ confocal microscope. The Coloc 2 plugin of Fiji was used to determine Pearson's coefficients of ROIs within background-subtracted images. Averages are reported +/− s.e.m.

### Cell Fluorescence and Membrane Protrusion Analysis

Images of MDA lines were acquired under the same microscope settings for calculations of mean protrusion fluorescence. All analysis was performed using Fiji. Maximum intensity projections of CD9 or CD81 z-stacks were thresholded until all cells were visible, and ROI outlines of cells created using the “analyze particles” function. Mean fluorescence and integrated density were measured within cell outlines that were overlayed onto original z-stacks. Mean protrusion fluorescence was calculated by subtracting the integrated density (mean fluorescence x area) of the cell body from the entire cell (which included protrusions), then dividing by the area difference: Mean protrusion fluorescence = (Int Dens total - Int Dens cell body)/(Area total – Area cell body). The z-slice containing the maximum fluorescence and its two surrounding z-slices were averaged for at least 4 XY positions to calculate mean protrusion and total fluorescence. ROI outlines of MDA-CD9-GFP, MA-11-CD9-GFP, and MCF-7-CD9-GFP lines were created as described above. Areas of protrusions were quantified by subtracting the area of the cell body from the total cell area for at least 3 XY positions. One-way ANOVA and Tukey post-hoc analysis were used to determine if there were statistically significant differences between BCC lines in terms of protrusion area and fluorescence. A minimum of 100 cells was evaluated for each condition.

### Scanning Electron Microscopy

BCCs and MDA CD9shRNA cells were plated and grown on coverslips coated with 0.01% poly-L-lysine (#P4707, Sigma, Darmstadt, Germany). After 2 days, they were fixed in 2% glutaraldehyde for 1 h at room temperature and then overnight at 4°C. Following 2-h post-fixation in 1% osmium tetroxide at 4°C, they were subjected to dehydration in an acetone gradient (25–100%) and critical point-dried in a CO_2_ system (Critical Point Dryer, Leica Microsystems, EM CPD 300, Wetzlar, Germany). Samples were then sputter-coated with gold (sputter coating device SCD 050; BAL-TEC GmbH, Witten, Germany) and examined at 5-kV accelerating voltage in field emission scanning electron microscope (Jeol JSM 7500F, Japan). The dimension (length and thickness) of various PMPs was quantified using ImageJ [60].

### *In Vivo* Studies

*In vivo* studies were performed by AntiCancer, Inc. upon approval by the AntiCancer Institutional Animal Care and Use Committee (IACUC) and conducted in accordance with the principles and procedures outlined in the National Institutes of Health Guide for the Care and Use of Animals under Assurance No. A3873-01. NU/NU nude mice from Charles River (http://www.criver.com/products-services/basic-research/find-a-model/nu-nu-nude-mouse) were kept within a barrier under HEPA filtration. and fed autoclaved laboratory rodent diet (Tecklad LM-485, Western Research Products, Orange, CA, USA). Viable MDA-DsRed and MDA-DsRed-CD9shRNA cells (6 × 10^6^) were resuspended in 100 μl 50% growth factor-reduced Matrigel and injected orthotopically into the second thoracic gland of 5-6 weeks old female *nu/nu* mice (10 animals/group). Mice were randomized based on age and weight. No blinding was done. Tumor size was measured by live-animal fluorescence imaging (FluorVivo) once per week and at study termination. Open imaging was performed to check metastases using the Olympus OV-100 imaging system (Olympus) at study termination. Mice were euthanized when the tumor burden exceeded 10 mm. in greater diameter. Euthanasia was achieved by 100% carbon dioxide inhalation, followed by cervical dislocation.

## SUPPLEMENTARY MATERIAL, FIGURES, TABLES











## Abbreviations

BCCs: breast cancer cells
MSCs: multipotent mesenchymal stromal cells
MDA: MDA-MB-231
PMPs: plasma membrane protrusions
HuMECs: human mammary epithelial cells
SEM: scanning electron microscopy
TIRF: total internal reflection fluorescence
MIP: maximum intensity projections
