# Supplementary material for: Tetraspanin CD9 determines invasiveness and tumorigenicity of human breast cancer cells
Source: Oncotarget. 2015 Feb 11;6(10):7970–91. doi: 10.18632/oncotarget.3419 (PMC4480729; doi:10.18632/oncotarget.3419)
Supplement: Supplementary file 5 [file oncotarget-06-7970-s005.pdf]

Tetraspanin CD9 determines invasiveness and tumorigenicity of human breast cancer cells

Supplementary Material

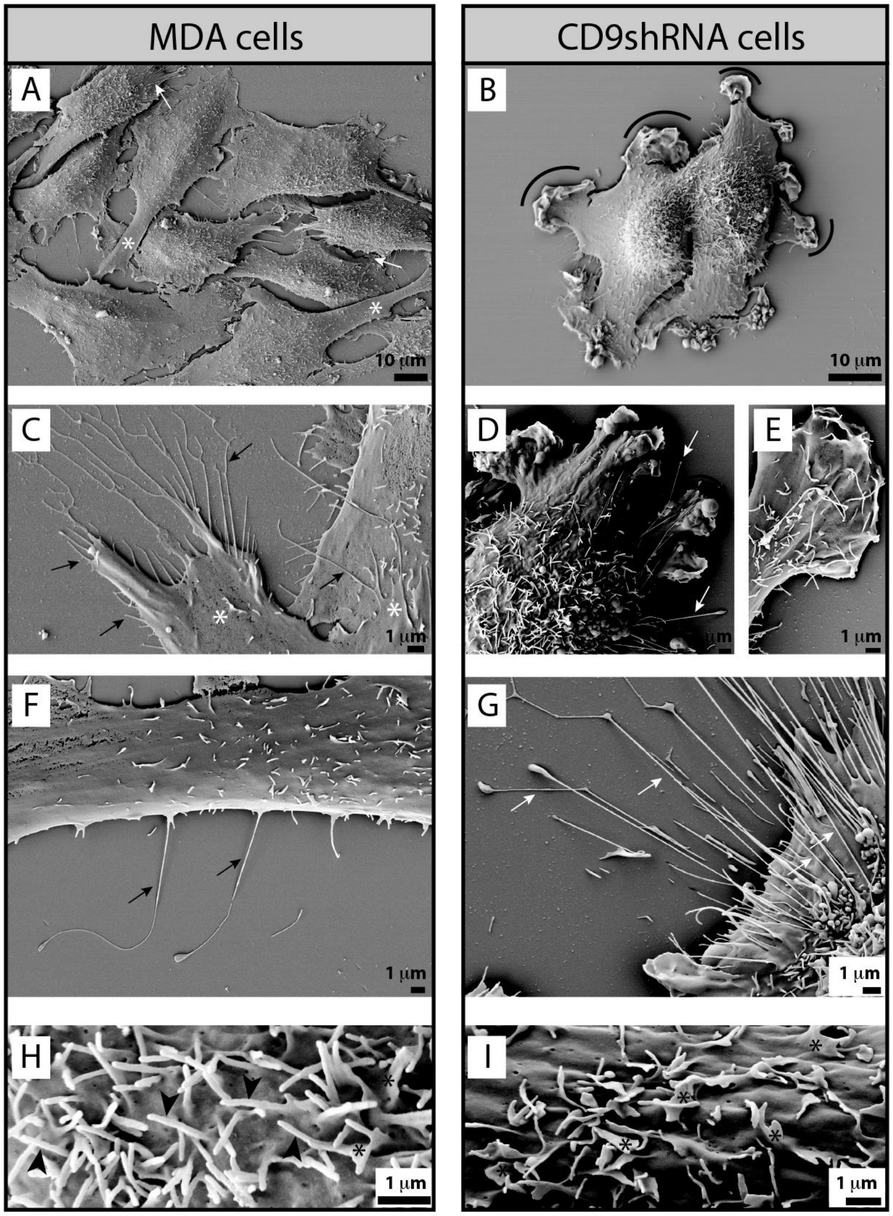

Figure S1: CD9 influences BCC morphology. A-I, Parental (A, C, F, H) and CD9-shRNA-transduced MDA (B, D, E, G, I) cells were analyzed by SEM. MDA cells develop lamellipodia (white asterisk), filopodia (black arrow), dorsal microvillus-like structures (black arrowhead) and very long and thick PMPs referred to as magnupodia (dashed arrow). In contrast, CD9-deficient cells have numerous membrane ruffles at their edge (arc) and dorsal part (black asterisk). Both cell lines develop thin membrane processes from their dorsal part (white arrow). Scale bars are indicated.

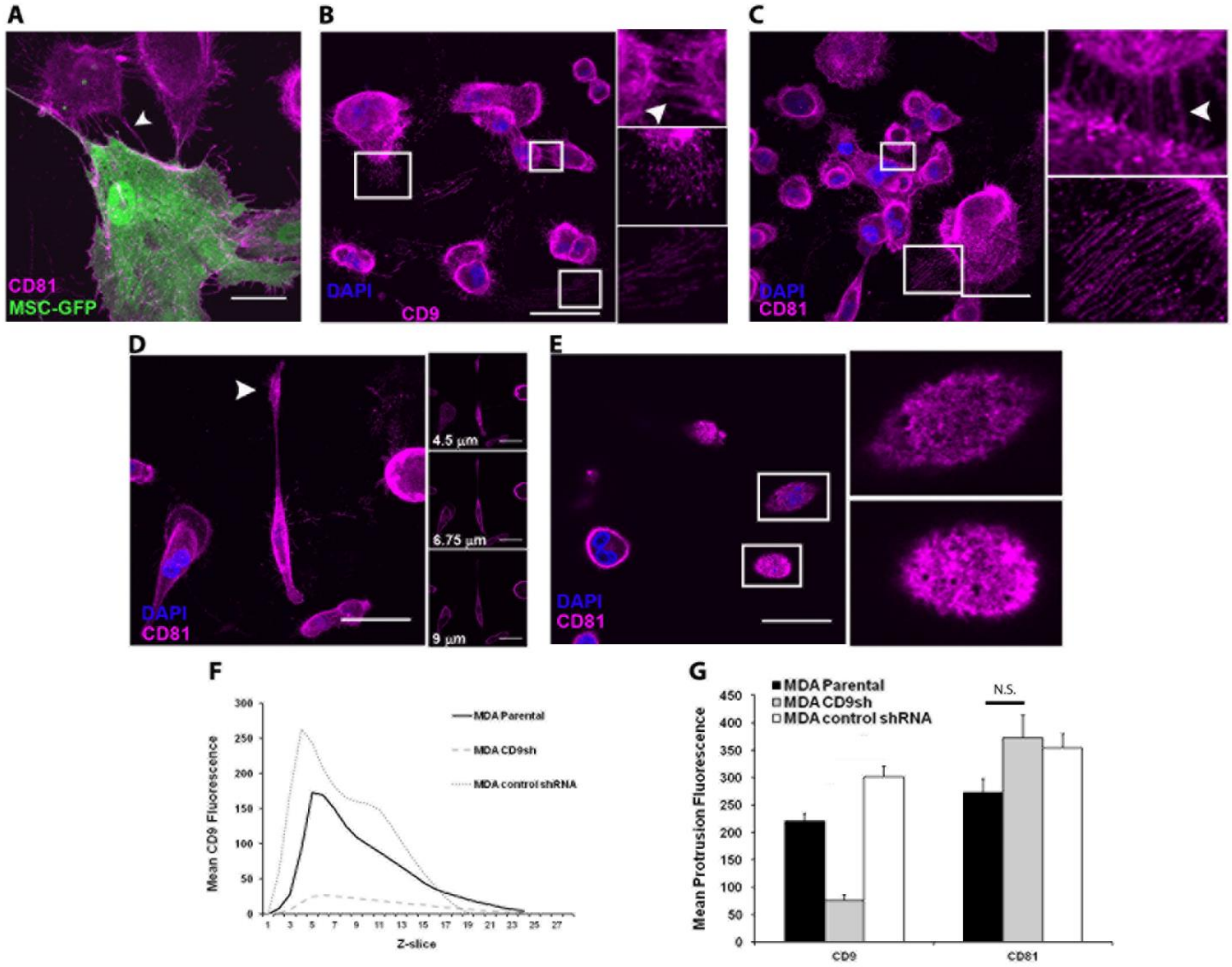

Figure S2: MDA control shRNA cells contain similar PMPs compared to parental MDA cells. A-E, MDA control shRNA cells (A-E) were cultured with GFP-transduced MSCs (A) or alone (B-E) prior their labeling with anti-CD81 (A, C-E) or anti-CD9 Ab (B). Arrowheads indicate thin filopodia emerging from a BCC interacting with a MSC (A), or short and thin processes connecting two MDA control shRNA cells (B, C) or magnupodium at the end of magnupodium (D). High power views of images of outlined areas are shown (B, C, E). MIP of a confocal z-stack with representative z-optical sections taken at different levels from the coverslip (D). Images are MIPs (A-D) or representative z-optical section (E). **F, G,** Mean of the total CD9 fluorescence along a confocal z-stack (F) and the mean protrusion CD9 and CD81 fluorescence (G) of MDA (parental), MDA CD9shRNA and MDA control shRNA cells. N.S., not significant. Scale bars, A, 25 µm, B-E, 50 µm.

Legends to Supplementary Videos

Supplementary Video S1: Representative TIRF time-lapse video showing plasma membrane interactions of MDA cells and MSCs. MDA-DsRed cells and MSCs-GFP were plated (1:1 ratio) at a density of 1,300 cells/cm^2 and incubated at 37°C overnight prior to the beginning of the experiment. TIRF images were recorded using 488 nm and 561 nm lasers over 12 h period. Scale bars, 25 µm.

Supplementary Video S2: Representative TIRF time-lapse video showing plasma membrane interactions of MDA CD9shRNA cells and MSCs. MDA-DsRed-CD9shRNA and MSCs-GFP cells were plated (1:1 ratio) at a density of 1,300 cells/cm^2 and incubated at 37°C overnight prior to the beginning of the experiment. TIRF images were recorded using 488 nm and 561 nm lasers over 12 h period. Scale bars, 25 µm.

Supplementary Video S3: Representative confocal time-lapse video showing full-cell interactions of MDA cells and MSCs. Compressed Z-stack images taken every 30 min of MDA-CD9-GFP and MSCs-DiI cells over 12 h period. Scale bars, 50 µm.

Supplementary Video S4: MSCs transfer materials to MDA cells. Transiently □-actin-mCherry transfected MSCs and MDA-CD9-GFP cells were plated (1:1 ratio) and incubated overnight. Afterward, TIRF images were taken every 15 min for 6 h period in a live-cell, temperature-controlled chamber. Scale bars, 25 µm.
